# When SEM becomes a deceptive tool of analysis: the unexpected discovery of epidermal glands with stalked ducts on the ultimate legs of geophilomorph centipedes

**DOI:** 10.1186/s12983-021-00402-3

**Published:** 2021-04-20

**Authors:** Andy Sombke, Carsten H. G. Müller

**Affiliations:** 1 Department of Evolutionary Biology, Integrative Zoology, University of Vienna, Althanstrasse 14, 1090 Vienna, Austria; 2 University of Greifswald, Zoological Institute and Museum, General and Systematic Zoology, Loitzer Straße 26, 17489 Greifswald, Germany

**Keywords:** Centipedes, Evolution, Histology, Ultrastructure, Functional morphology

## Abstract

**Background:**

The jointed appendage is a key novelty in arthropod evolution and arthropod legs are known to vary enormously in relation to function. Among centipedes, the ultimate legs always are distinctly different from locomotory legs, and different centipede taxa evolved different structural and functional modifications. In Geophilomorpha (soil centipedes), ultimate legs do not participate in locomotion and were interpret to serve a sensory function. They can be sexually dimorphic and in some species, male ultimate legs notably appear “hairy”. It can be assumed that the high abundance of sensilla indicates a pronounced sensory function. This study seeks for assessing the sensory diversity, however, documents the surprising and unique case of an extensive glandular epithelium in the ultimate legs of three phylogenetically distant species.

**Results:**

The tightly aggregated epidermal glands with stalked ducts – mistakenly thought to be sensilla – were scrutinized using a multimodal microscopic approach comprising histology as well as scanning and transmission electron microscopy in *Haplophilus subterraneus*. Hence, this is the first detailed account on centipede ultimate legs demonstrating an evolutionary transformation into a “secretory leg”. Additionally, we investigated sensory structures as well as anatomical features using microCT analysis. Contrary to its nomination as a tarsus, tarsus 1 possesses intrinsic musculature, which is an indication that this podomere might be a derivate of the tibia.

**Discussion:**

The presence and identity of ultimate leg associated epidermal glands with stalked ducts is a new discovery for myriapods. A pronounced secretory as well as moderate sensory function in *Haplophilus subterraneus* can be concluded. The set of characters will improve future taxonomic studies, to test the hypotheses whether the presence of these specialized glands is a common feature in Geophilomorpha, and that tarsus 1 may be a derivate of the tibia. As the number of epidermal glands with stalked ducts is sexually dimorphic, their function might be connected to reproduction or a sex-specific defensive role. Our results, in particular the unexpected discovery of ‘glandular hairs’, may account for a striking example for how deceptive morphological descriptions of epidermal organs may be, if based on non-invasive techniques alone.

## Background

**Fig. 1:**
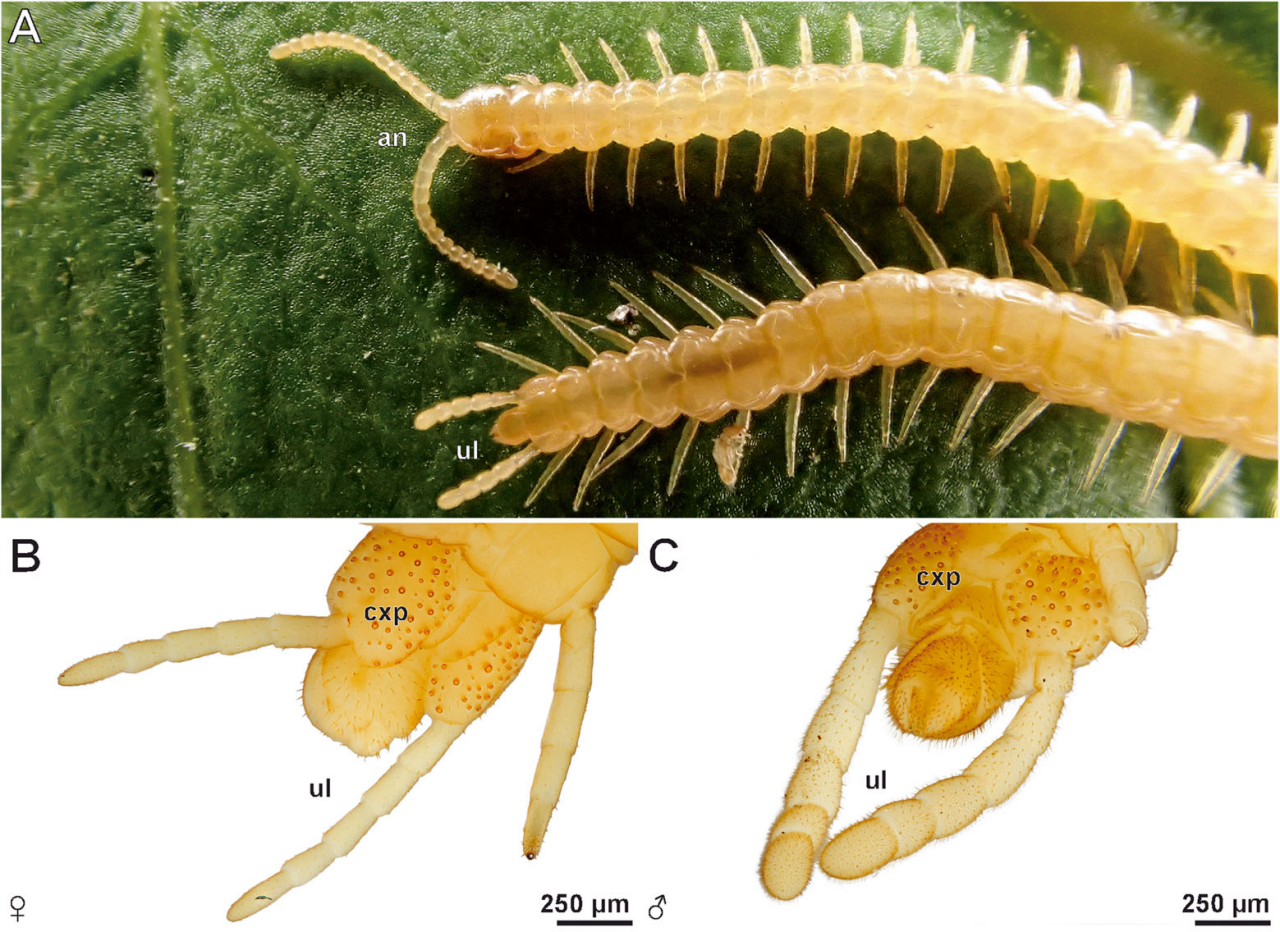
The geophilomorph centipede *Haplophilus subterraneus*. **a** Male specimen with focus on head and posterior trunk. Note the morphological disparity of locomotory and ultimate legs. **b** Female posterior trunk with ultimate legs, view from ventral (locomotory legs partially omitted). **c** Male posterior trunk with ultimate legs, view from ventral (locomotory legs partially omitted). Abbreviations: **an** antennae, **cxp** coxal pores, **ul** ultimate legs

The jointed arthropod appendage – the arthropodium – consisting of podomeres interconnected by flexible joints, is a key novelty in the evolutionary history of arthropods. It shows an enormous degree of structural and functional variations. The entity of arthropodia attached to an arthropod body not only enables locomotion, but also allows for many other essential biological functions like food capture and manipulation, cleaning, mating, or sensing the environment. As the arthropod cuticle is mostly rigid and impermeable for a vast number of chemicals, the transduction of mechanical, chemical, and other stimuli is achieved by modified ciliary exteroreceptors (except photoreception), which are usually confined to trichoid or peg-like sensilla. These trichoid sensilla are easily recognizable externally by their sensory projection – the sensillum shaft (e.g. see reviews by [1–3]), and it is the sensory and locomotory appendages, in particular, that are riddled with these sensilla. Among the five extant subtaxa of centipedes, locomotory legs are widely uniform, but considerable variation does occur at both ends of the body. Besides the antennae, the last pair of legs, called ‘ultimate legs’, exhibits a particular variety of morphologies and functions [4]. Different centipede taxa evolved different structural and functional modifications of ultimate legs: while scutigeromorph centipedes (house centipedes) always bear elongated, antenna-like ultimate legs, their morphology and function varies considerably among pleurostigmophoran taxa, such as Lithobiomorpha or Scolopendromorpha [4–6]. As there is a high degree of interspecific or -generic disparity in ultimate leg morphology, a functional differentiation must be assumed. Based on their resemblance to antennae, scutigeromorph ultimate legs may be considered as sensory appendages, while ultimate legs in some lithobiomorph species play a major role in predator avoidance by secretion of sticky substances [7, 8]. Extremely conspicuous modifications do occur e.g., in the scolopendromorph genus *Alipes* (flag tail centipedes), in which ultimate legs are leaf-like and used for stridulation [9]. Within the taxon Geophilomorpha (soil centipedes), however, ultimate leg modifications appear less spectacular. It can be easily observed that the last legs do not participate in locomotion and as centipedes are capable of moving backwards (especially soil living geophilomorphs), it was proposed that they serve a sensory function at the posterior end of the body [4, 10, 11]. In contrast to locomotory legs, geophilomorph ultimate legs are oriented along the longitudinal body axis (pointing backwards; Fig. 1a) and in many cases appear swollen or slightly bigger (e.g. [12–14]). In some taxa, the claw is reduced and the ultimate legs might possess a higher abundance of cuticular structures, like trichomes or sensilla (they notably appear “hirsute”). Among others, these characteristics are considered relevant for species identification [12, 13, 15]. Based on previous research on the sensory abilities of centipede ultimate legs, it can be assumed that the high abundance of putative sensilla may indicate a pronounced sensory function [4, 6, 11, 16]. This indication may be further substantiated by earlier descriptions on the chemo- and mechanoreceptive properties of ultimate legs in geophilomorphs. Rajulu [11] analyzed the anatomy of ultimate legs in *Himantarium samuelraji* and conducted electrophysiological experiments showing that presumptive sensory organs located on the ultimate leg tarsi are capable of detecting chemical stimuli. In addition, geophilomorph ultimate legs can exhibit sexual dimorphisms [12, 17]. Accordingly, also intraspecific variations occur. However, nothing is known about the quality and variety of senses covered by geophilomorph ultimate legs with a particularly higher number of putative sensilla. At least, their involvement in mate recognition or spermatophore placement was assumed based on behavioral observations [18, 19].

The present study seeks for assessing the functional diversity of ultimate legs in centipedes in general and of geophilomorphs in particular. We here, however, document the surprising and unique case of an extensive glandular epithelium in the ultimate legs of three phylogenetically distant species of Geophilomorpha, among them the common northwestern European species *Haplophilus subterraneus*. The tightly aggregated gland units with stalked ducts – mistakenly thought to be sensilla – were scrutinized using a multimodal microscopic approach comprising paraffin and semithin-sectioning histology as well as scanning and transmission electron microscopy. The ultrastructure of the glandular units is compared to those of other aggregated epidermal glands so far known from centipedes, and similarities and variations are discussed with respect to their evolution. We furthermore address critical aspects of studies exclusively relying on non-invasive methods and what may be inferred from our findings for future studies targeting the diversity, systematic, and evolution of centipedes.

## Results

### External morphology of the ultimate legs – sexual dimorphisms and specialized epidermal glands

**Fig. 2:**
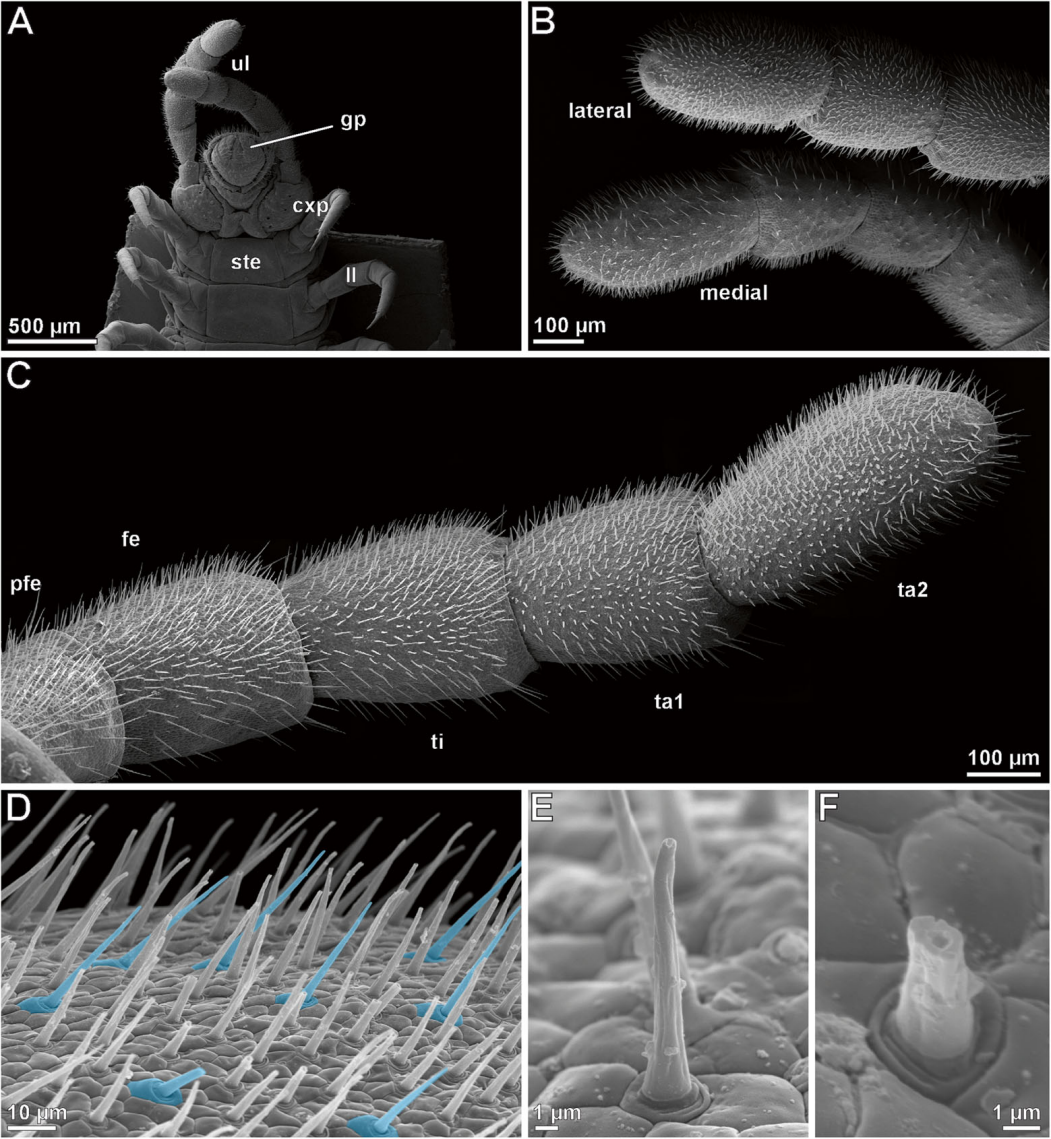
SEM analysis of the ultimate legs of male *Haplophilus subterraneus*. **a** Posterior trunk from ventral. Note the size and insertion of ultimate legs in comparison to locomotory legs. The enlarged coxae bear several coxal pores. **b** Ultimate legs in ventrolateral view. Medial sides exhibit far less stalked ducts from epidermal glands then lateral and dorsal sides. **c** Lateral side of male ultimate leg (different specimen then (**a** and **b**)). Stalked ducts of epidermal glands and trichoid sensilla cover the whole surface. **d** Magnification of the tibia. Trichoid sensilla are highlighted in blue and noticeable by their characteristic socket (compare Fig. 4b) and a lower angle to the leg cuticle. **e** Stalked duct of an epidermal gland. Note the characteristic round socket that is completely encompassed by scutes (epidermal cell profiles), and the large terminal pore. **f** Stalked duct broken off slightly above the socket region. Note the duct visible as central canal surrounded by the stalk wall cuticle, and also the round socket. Abbreviations: **cxp** coxal pores, **fe** femur, **gp** gonopods, **ll** locomotory leg, **pfe** prefemur, **ste** sternite, **ta1** tarsus 1, **ta2** tarsus 2, **ti** tibia, **ul** ultimate leg

**Fig. 3:**
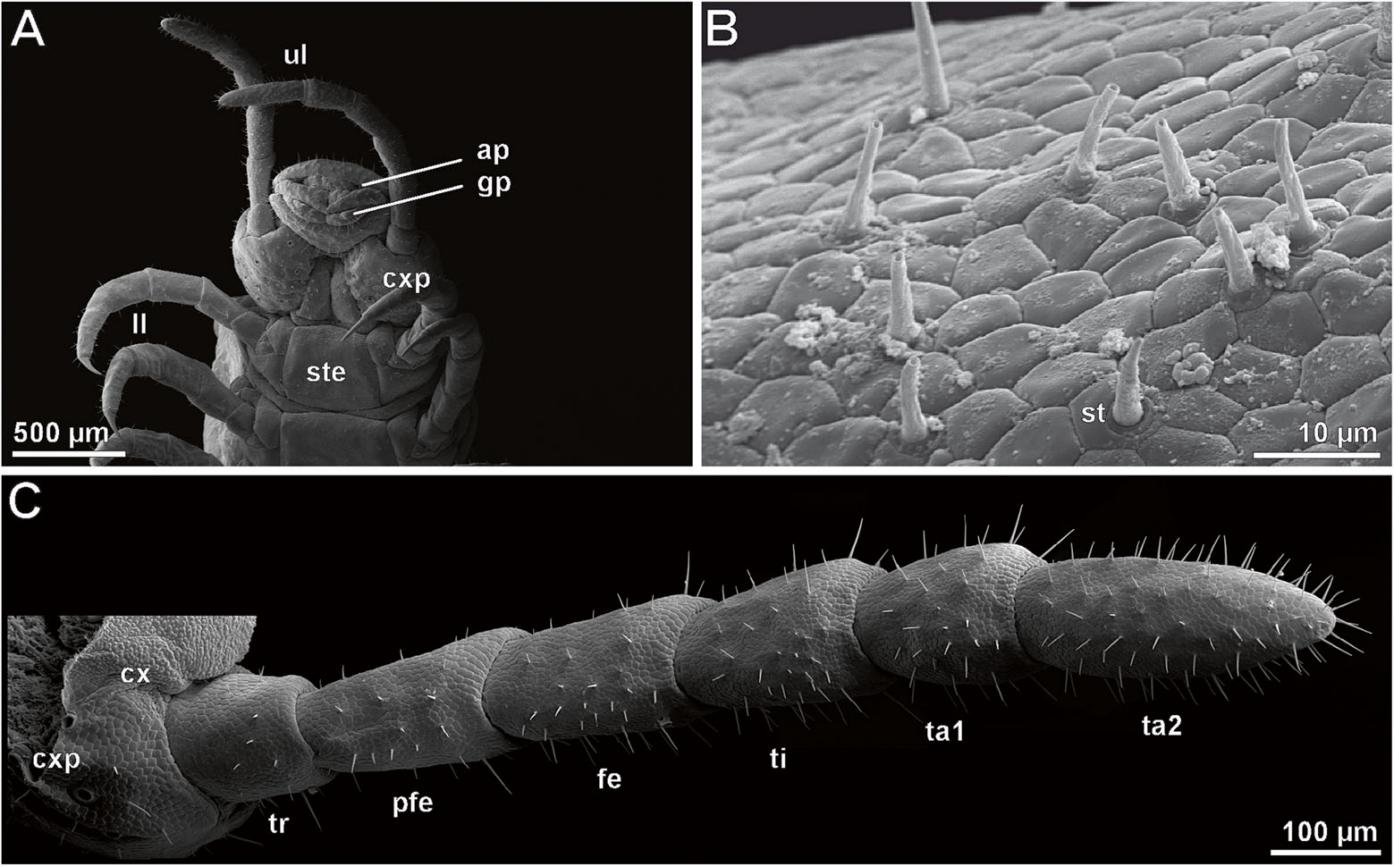
SEM analysis of the ultimate legs of female *Haplophilus subterraneus*. **a** Posterior trunk from ventral. Note the shape and insertion of ultimate legs in comparison to locomotory legs. **b** Stalked ducts of epidermal glands scattered on the tibia. Note also the trichoid sensillum in the lower center (st; with characteristic socket). **c** Ventromedial side of female ultimate leg. Abbreviations: **ap** anal pore, **cx** coxa, **cxp** coxal pores, **fe** femur, **gp** gonopods, **ll** locomotory leg, **pfe** prefemur, **st** sensillum trichodeum, **ste** sternite, **ta1** tarsus 1, **ta2** tarsus 2, **ti** tibia, **ul** ultimate leg

Macrophotography and SEM analyses of female and male ultimate legs in *Haplophilus subterraneus* revealed that their external morphology differs from locomotory legs (compare Figs. 1a versus b and c and 2a versus 3a), and that there is sexual dimorphism in size and proportions (Fig. 1b, c). In comparison to locomotory legs, ultimate legs in both sexes possess two tarsi, lack a claw (pretarsus), and exhibit a large coxa with several coxal pores/organs (e.g. Figs. 1b and 2a; cxp). Male ultimate legs are distinctly thicker than female ones (compare also Fig. 1b vs c). In males, each ultimate leg is heavily covered by cuticular structures representing sensilla and epidermal glands with stalked ducts (Fig. 2b–d). However, in low magnification, differences and abundances of trichoid sensilla and epidermal glands with stalked ducts are not or hardly distinguishable – only at high magnification, differences are evident (see below). In males, the abundance of both types of cuticular structures is ca. 4000 (ca. 150 on prefemur, 450 on femur, 600 on tibia, 1000 on tarsus 1, and 1500 on tarsus 2; *N* = 2). In general, the abundance of both cuticular structures is much lower on medial faces (Fig. 2b). In females, the abundance of sensilla and epidermal glands with stalked ducts is ca. 450 (ca. 20 on prefemur, 30 on femur, 50 on tibia, 100 in tarsus, and 250 on tarsus 2; *N* = 2). Likewise, the medial faces are nearly free of both types of cuticular structures (Fig. 3d, g). In both sexes, tarsus 2 exhibits a small terminal invagination (Fig. 5a-c).

#### Epidermal glands with stalked ducts

**Fig. 4:**
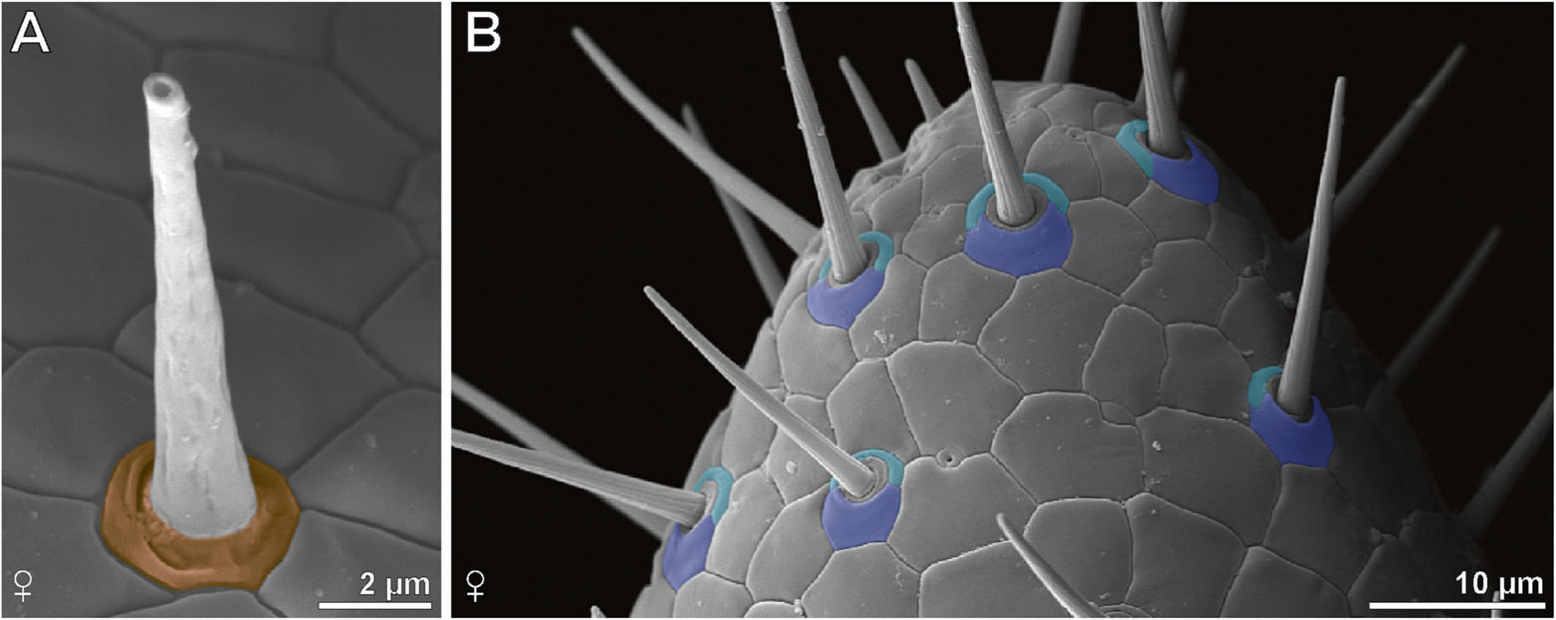
Morphological features used to distinguish epidermal glands with stalked ducts from trichoid sensilla as depicted from SEM. **a** Gland stalk in female *H. subterraneus* with colored round socket and large terminal pore. The socket exhibits one or two cuticular folds (compare left top and right bottom) that merge with the shaft cuticle. The shaft is irregularly notched. **b** Tip of female tarsus 2 with several trichoid sensilla. The socket is always composed of two scutes: a larger polygonal scute distally (darker blue) and a sickle-shaped scute proximally (lighter blue). The shaft bears ribs, which are strictly proximally and slightly bent distally to form a steep spiral, a small terminal pore is present (not visible in this image)

Based on SEM analyses, the majority of cuticular structures on the male ultimate leg are shaft-like protuberances. As they resemble a stalk, we address these organules as ‘epidermal glands with stalked ducts’. In this description, the term ‘gland stalk’ is used as a synonym for a stalked duct if the entirety or only the outer appearance of the shaft-like protuberance is addressed. At the tip of the gland stalk, a large terminal pore (300–500 nm in diameter) is present (Figs. 2e and 4a). In some aspects, these stalks strongly resemble the shafts of neighboring trichoid sensilla, but can be distinguished by their socket that exhibits one or two cuticular folds that merge with the cuticle (Figs. 2e, f, 3b and 4a). In addition, these sockets are in direct contact with epidermal scutes, which indicate the subcuticular arrangement of epidermal cells [20]. In males, one to two scutes are present between the stalks (Fig. 2d, e); in females, the distance can be wider (Fig. 3b). The stalked ducts of epidermal glands exhibit a 60–70° angle relative to the leg’s cuticle, which make them additionally distinguishable from trichoid sensilla that project from the cuticle in a lower angle (blue in Fig. 2d). The irregularly notched stalked ducts are approximately 15 μm long in males and approx. 8 μm long in females (*n* = 20). Broken off stalks reveal a central canal (Fig. 2f). In males, approximately 3500 stalked ducts of epidermal glands are present on each ultimate leg (*N* = 2); in females their abundance is about 100–150 (*N* = 2) (Figs. 2b–d and 3b, c). Thus, their ratio to sensilla is roughly 7:1 in males (Fig. 2d) as compared to 1:2 in females.

#### Sensillum trichodeum

In males and females, ca. 300–500 sensilla trichodea occur on each ultimate leg (less in females). In females, tarsus 1 and 2 are mostly associated with trichoid sensilla (Fig. 3c). In detail, s. trichodea can be distinguished from stalked ducts of epidermal glands by their socket structure and the lower angle of the shaft in relation to the leg’s cuticle (Fig. 2d). The movable socket is always encompassed by two scutes: a larger polygonal scute distally, and a sickle-shaped scute proximally (Fig. 4b). The shaft is vertically ribbed and 8–20 μm in length. A small terminal pore is present (not shown).

#### Sensillum microtrichodeum

**Fig. 5:**
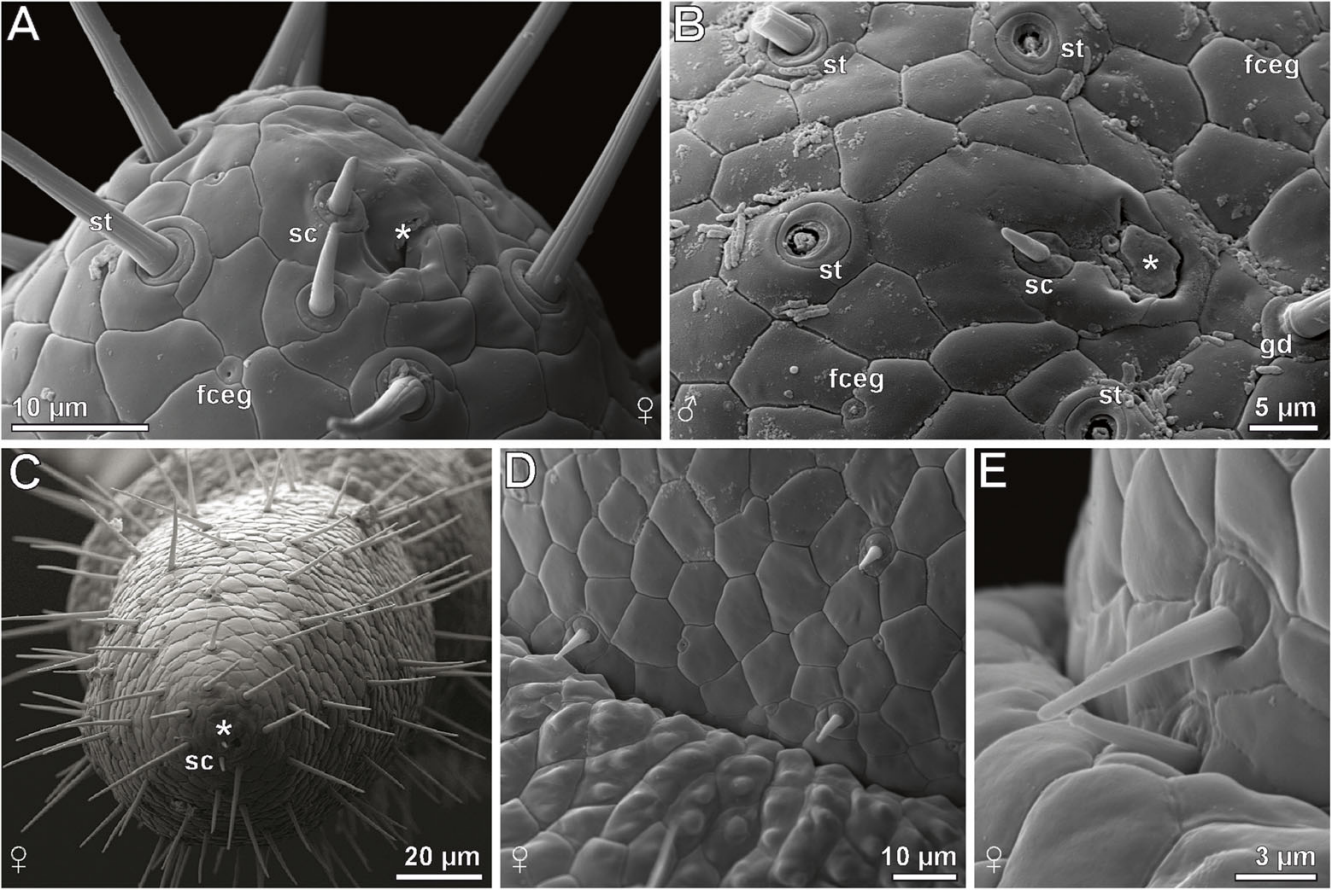
Details of the ultimate leg tip and podomeres in female and male *Haplophilus subterraneus* as depicted from SEM. **a** Tip of tarsus 2 of a female’s ultimate leg, which is encircled by trichoid sensilla (note the characteristic socket). Encompassed by that circle, 1–2 sensory cones with round socket are present, located close to a depression on a larger cuticular field (asterisk). Frequently, smaller pores of solitary flexo-canal glands are present. **b** Tip of tarsus 2 in a male ultimate leg. Likewise, one or two sensory cones framed by a larger cuticular field and flanked by a fissured area of the cuticle are present (asterisk; obscured by secretion or dirt). Several shafts of trichoid sensilla are broken off. **c** Apical view on the female tarsus 2 tip with terminal invagination (asterisk), two sensory cones, and mostly trichoid sensilla (which are hard to distinguish at this magnification and viewing angle). **d** Sensilla microtrichodea at the transition of tibia and femur in a female ultimate leg. **e** Detail view of two sensilla microtrichodea at the transition of tibia and femur. Note the deflection of the lower sensillum and the restricting socket in the upper sensillum. Abbreviations: **fceg** solitary flexo-canal gland, **gd** stalked duct of epidermal gland, **sc** sensory cone, **st** sensillum trichodeum

These sensilla are present at and near the transitions of ultimate leg podomeres in males and females (Fig. 5d, e). The shaft is about 5–8 μm in length, a small terminal pore is present. The socket resembles that of longer sensilla trichodea, with a movable part and two characteristic scutes (Fig. 5e).

#### Sensory cones

At the tip of tarsus 2, one or two small cone-like sensilla are present (in males and females). The short shaft is ca. 3 μm in length, possesses a rounded tip, and lacks terminal or wall pores. A single cuticular ring with a tangential groove surrounds the shaft base (Fig. 5a, b; sc). The sensory cone in terminal position always is located on a larger scute associated with a depression/invagination (Fig. 5a, b).

#### Flexo-canal epidermal glands

Frequently, small pores of flexo-canal epidermal glands (for classification see [21]) are present on the ultimate legs. They are never elevated and always located between the scutes, and have a diameter of approx. 1.5 μm. The diameter of the pore is about 0.2 μm (Fig. 5a, b; fceg).

### Anatomy of the ultimate leg

**Fig. 6:**
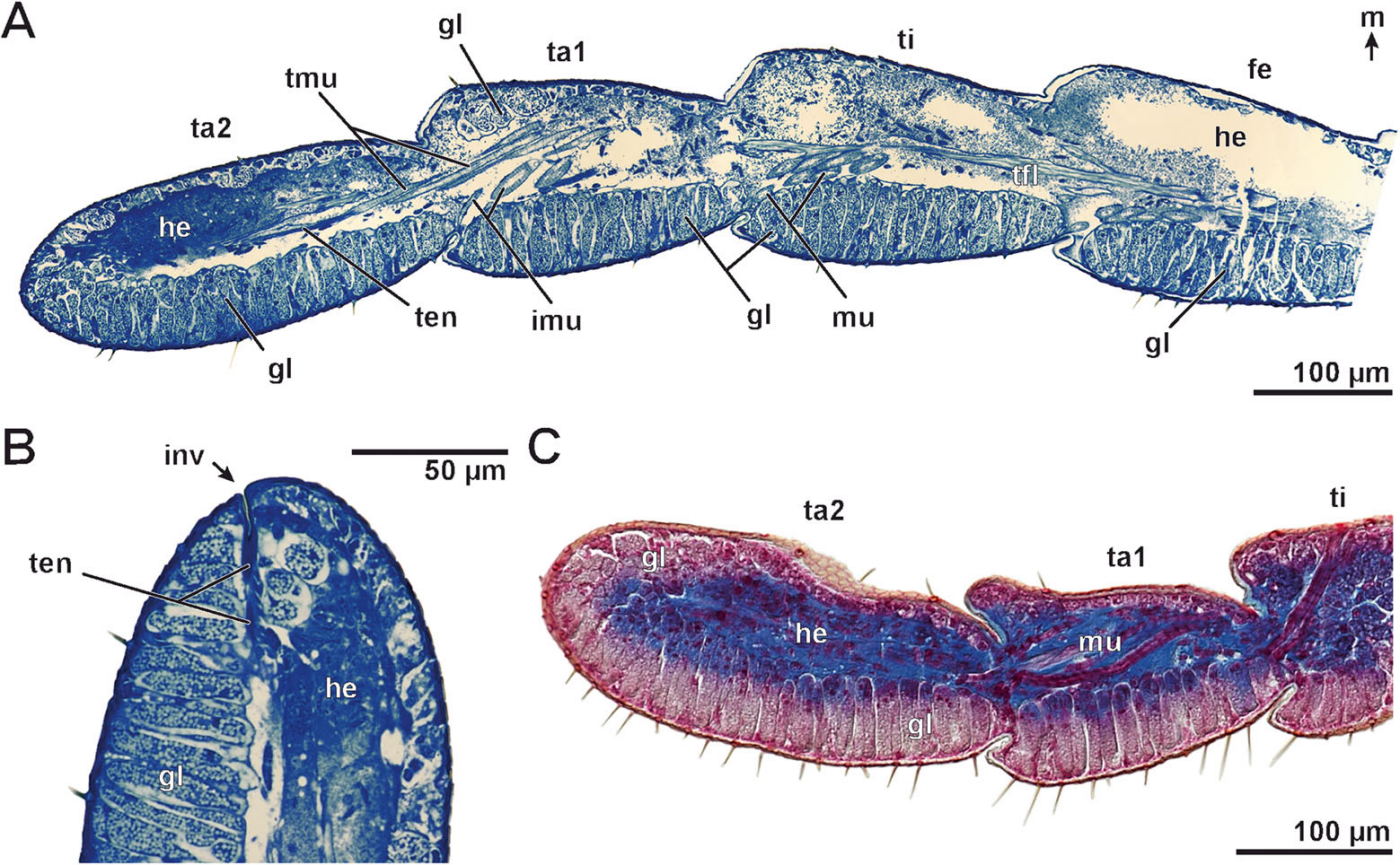
Anatomy of ultimate legs in male *Haplophilus subterraneus* from semi-thin (**a** and **b**) and paraffin (**c**) sections. **a** Longitudinal section of the telopodite of an ultimate leg (composite), dorsomedial side to the top. The leg’s epidermis displays a massively developed glandular tissue: at the bottom (lateral to ventrolateral), secretory cells of mostly epidermal glands with stalked ducts are visible in sagittal section plane; whereas towards the top (medial to dorsomedial), secretory cells are cross-cut. The tendon and associated musculature traverse podomeres. Tarsus 1 possesses intrinsic musculature that terminates in the distal joint region of tarsus 1. Other muscles in tarsus 1 contribute to the tendon musculature (compare also Fig. 7d, e). Frequently, coagulated hemolymph is heavily stained (especially in tarsus 2). **b** Detail view of the distal tip of tarsus 2. This section reveals that the externally visible notch/depression (compare Fig. 5a-c) continues approx. 10 μm and connects with the tendon. **c** Sagittal section of the distal podomeres of the ultimate leg. Likewise, sagittal and cross section profiles of secretory cells of epidermal glands are visible (compare **a**). In this preparation, the association of stalked ducts and epidermal glands is evident. The cytoplasm of the glands is stained pale pink, nuclei and musculature red, and the hemolymph blue. Abbrevtiations: **fe** femur, **imu** intrinsic musculature of tarsus 1, **inv** terminal invagination, **gl** glandular tissue, **he** hemolymph, **m** medial, **mu** musculature, **ta1** tarsus 1, **ta2** tarsus 2, **ten** tendon, **ti** tibia, **tfl** tendon flexor, **tmu** tendon musculature

**Fig. 7:**
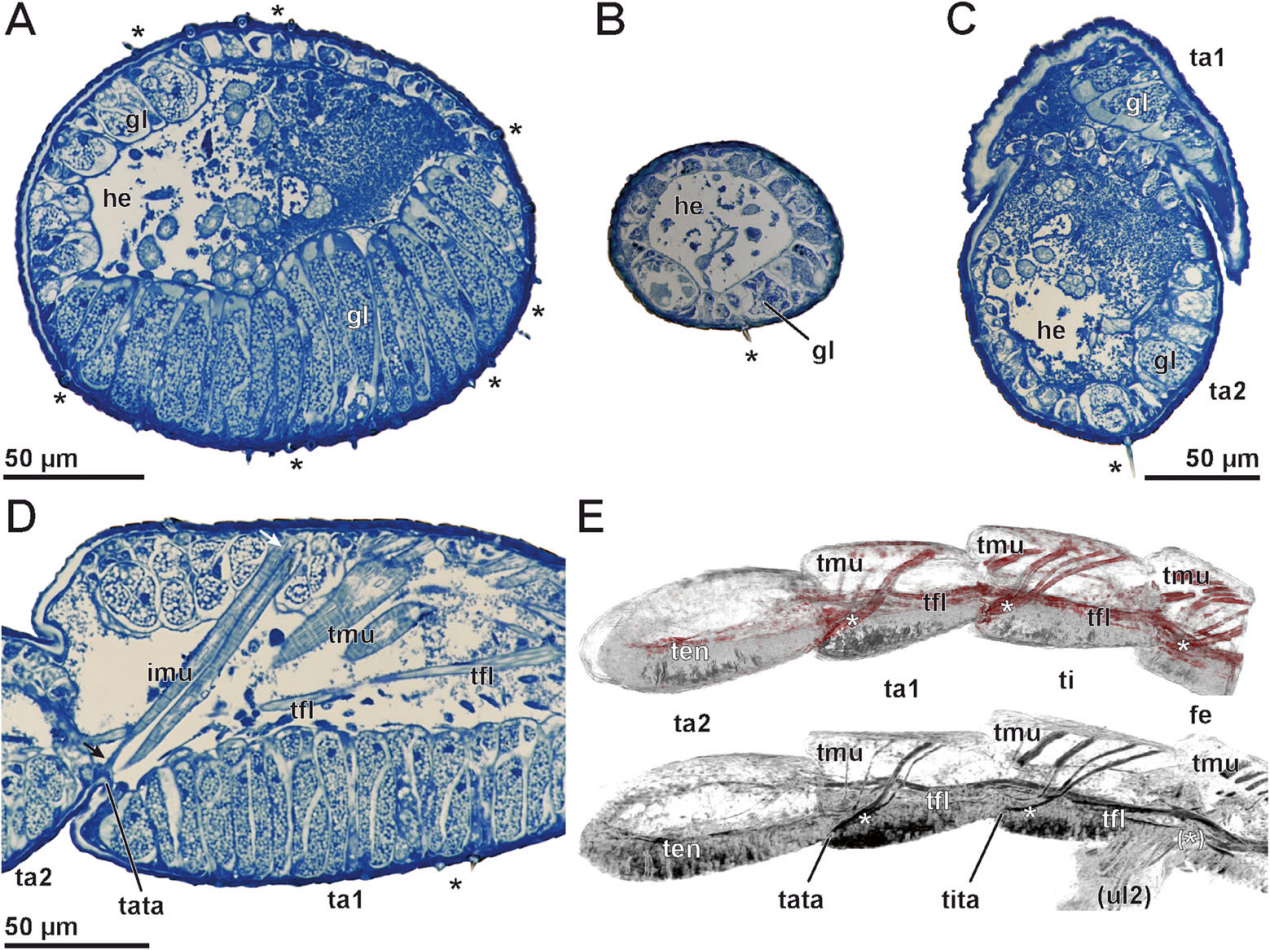
Anatomy of ultimate legs in female and male *Haplophilus subterraneus* from semithin sections (**a**-**d**) and microCT analysis (**e**). **a** Cross section of a male tarsus 2 (midsection), to scale with female tarsus 2 (**b**). The polarity of glandular tissue is evident. Secretory cells of epidermal glands with stalked ducts are lined up in a palisade-like fashion (bottom). In the top of the section, secretory cells of the same gland type are visible in cross-section. **b** Cross section of a female tarsus 2 (midsection). Only few secretory cells of epidermal glands are present. **c** Oblique cross section of ultimate leg (male) at the transition of tarsus 2 to tarsus 1. The glandular tissue lines most of the leg and continues from one podomere to the next. Note the stalked ducts of epidermal glands (asterisk). **d** Tarsus 1 of the ultimate leg (male), exhibiting glandular tissue and intrinsic musculature spanning from the top cuticle to the tarsus1-tarsus2-joint (arrows). Larger muscles (top right) attach to the tendon. **e** MicroCT analysis of ultimate leg musculature (male specimen). Due to similar tissue densities, musculature and glandular tissue are hardly separable, but in both visualizations (top: Drishti, bottom: Amira), the tendon musculature/flexors (tfl) are detectable. From the top cuticle of femur, tibia and tarsus 1 stronger muscle projects to the distal podomere border, respectively. Tendon musculature (tmu) attaches do the tendon. Intrinsic musculature (asterisks) attached ventrodistally to the following podomeres. Due to different specimen and viewing angle, musculature trajectories may vary. Tarsus 2 is devoid of intrinsic musculature. In the lower visualization, parts of the second ultimate leg are visible (ul2). Abbrevtiations: **fe** femur, **gl** glandular tissue, **he** hemolymph, **imu** intrinsic musculature, **ta1** tarsus 1, **ta2** tarsus 2, **tata** tarsus1-tarsus2-border, **ti** tibia, **tita** tibia-tarsus1-border, **tfl** tendon flexor, **tmu** tendon musculature

Paraffin and semi-thin sections were used to reveal the anatomy of the gland stalks as well as of the ultimate leg podomeres. In males, where stalked ducts of epidermal glands and sensillar shafts cover the large ultimate legs intensely, a massively developed glandular tissue in all ultimate leg podomeres fills up the leg lumen (Figs. 6 and 7a, c, d). Large secretory cells are lined in a palisade-like fashion at dorsal, lateral and ventral sides of all ultimate leg podomeres (Figs. 6 and 7a, c, d; gl). In some sections, the association of stalked ducts and the subcuticular domains of epidermal glands is evident (Fig. 6). Female ultimate legs are far thinner than male ones (female ca. 80–100 μm, Fig. 7b; male ca. 180–200 μm; Fig. 7a). Concerning sexual dimorphism, the cuticle of the female ultimate leg is only sparsely riddled with gland ducts (Fig. 7b). A palisade-like arrangement of secretory cells is absent (compare Fig. 7a, b). In females, secretory cells of epidermal glands are smaller than those present in males. The ‘glandular tissue’ is more or less continuous in male ultimate legs across the proximal and distal borders of given podomere (Fig. 7c) – hence, the tight arrangement of glandular modules (for details see below) identifies them as ‘aggregated epidermal glands’.

The center of ultimate leg podomeres is filled with hemolymph (partially heavily stained in histological sections; Fig. 6; he), as well as musculature and the tendon (claw tendon) (Figs. 6 and 7d, e). As the claw is absent, the tendon attaches to a thin apodeme (approx. 10 μm deep) at the terminal tarsus 2 (Fig. 6b; ten). The apodeme is indicated by the small invagination at the tip of tarsus 2 (compare asterisks in Figs. 5a–c and 6b). Onto the tendon, which traverses several podomeres, several tendon flexors attach (Figs. 6a, c and 7d, e; tmu). In histological sections and microCT analysis (that gives additional insights into podomere anatomy), the tendon is, however, not well detectable (Figs. 6a and 7d, e). Muscles that attach to the tendon originate in tarsus 1, tibia, femur, and prefemur (tendon flexors). In addition, intrinsic muscles attach ventrodistally to the following (distal) podomeres (asterisks in Fig. 7e; imu), projecting from the dorso-proximal part of tarsus 1, tibia, and femur (Figs. 6a, c and 7d, e; imu). Thus, also tarsus 1 possesses intrinsic flexure musculature. Tarsus 2 is devoid of intrinsic musculature.

### Ultrastructure of ultimate leg associated epidermal glands and trichoid sensilla

**Fig. 8:**
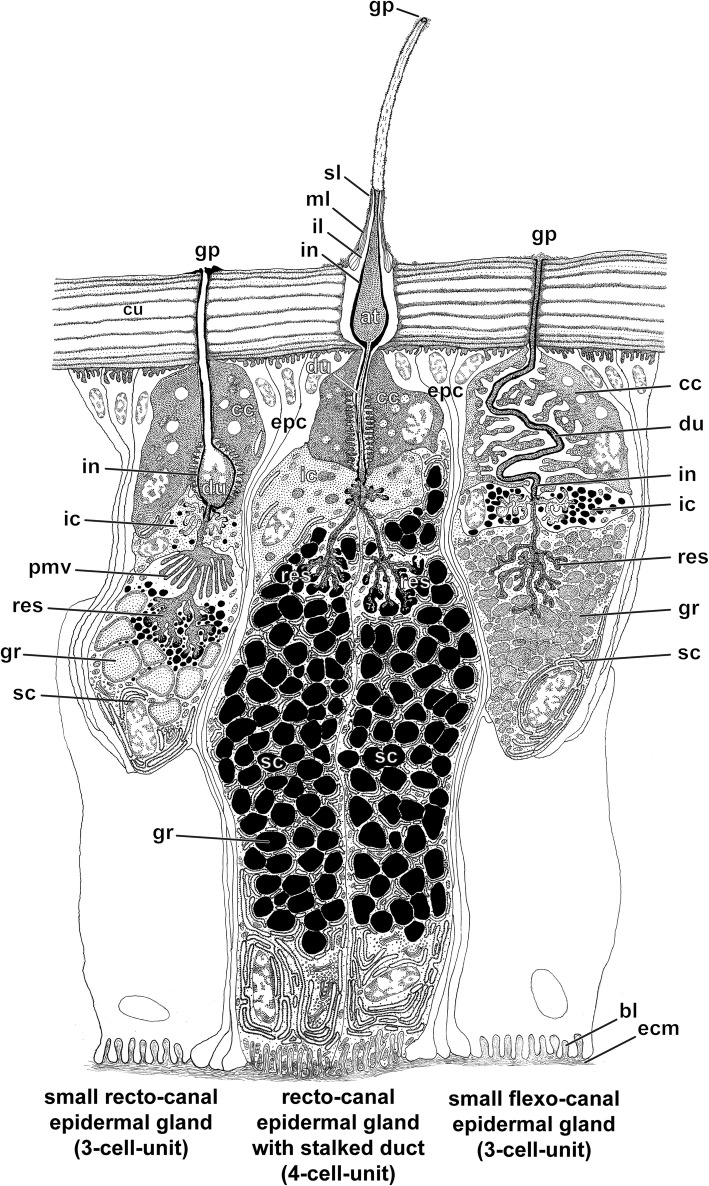
Cellular organization of three types of epidermal glands common in the glandular epithelium of the ultimate leg of *Haplophilus subterraneus* in longitudinal view. The proximal part of the stalked duct/gland is cut longitudinally, whereas its distal and median parts are shown in full to demonstrate the terminal (gland) pore (gp) and secretion discharged the remains of which potentially establish the extremely electron-dense outer layer (sl). Secretory cells of neighboring epidermal glands with stalked ducts are sketched without detail illustration of cytoplasmic content. Abbreviations: **at** atrium, **bl** basal labyrinth, **cc** canal cell, **cu** leg cuticle, **du** duct, **ecm** extracellular matrix, **epc** interstitial epidermal cells, **gr** secretory granule, **ic** intermediary cell, **il** inner fibrous layer, **in** cuticular intima, **ml** median lamellated layer, **pmv** palisade-like formation of microvilli, **res** reservoir, **sc** secretory cell

**Fig. 9:**
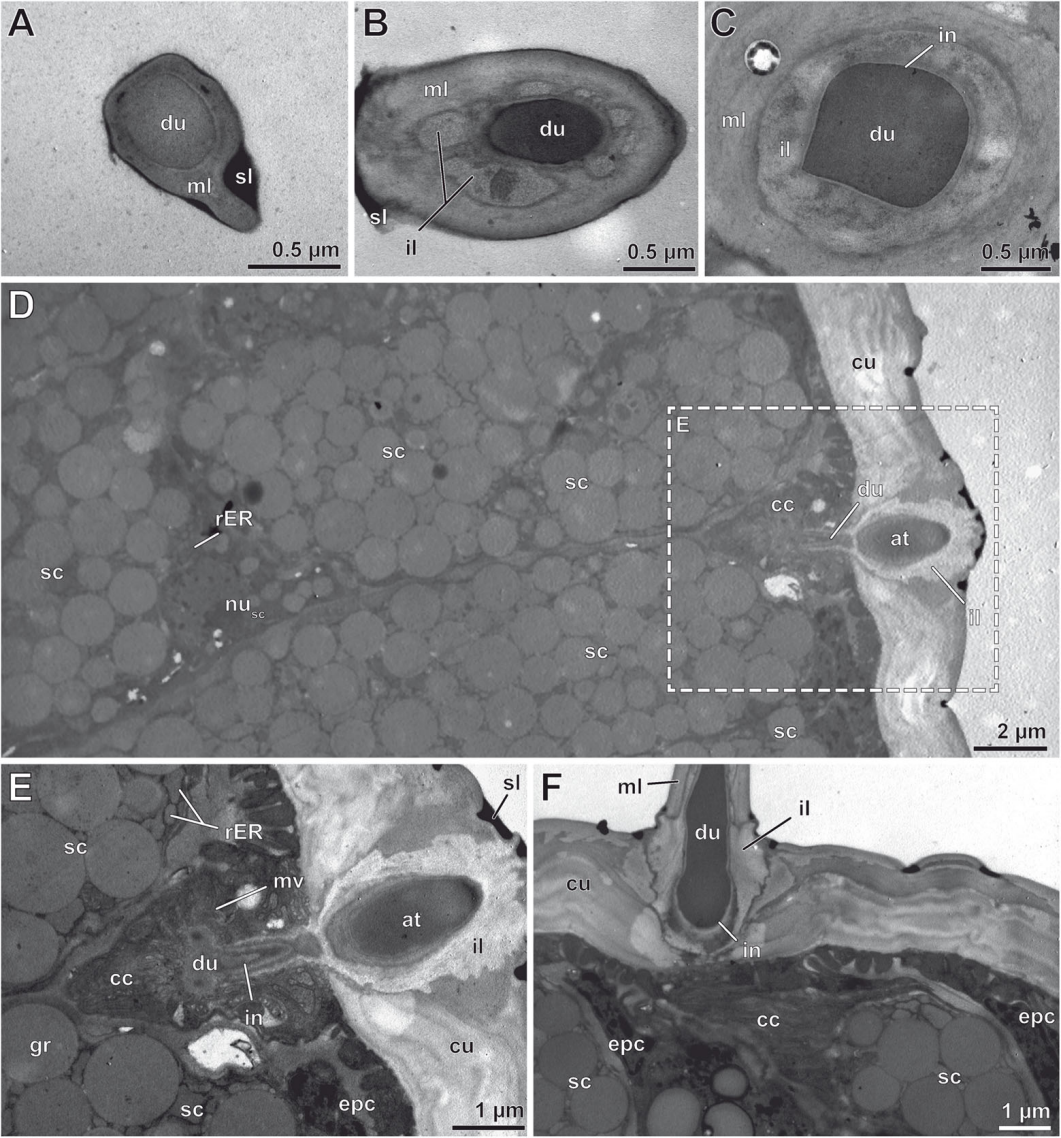
Ultrastructure of epidermal glands with stalked ducts located on the male ultimate legs of *Haplophilus subterraneus* as depicted from TEM. Stalked duct and canal cell. **a**-**c** Cross sections at various levels of the gland stalk including the extended duct (filled with highly osmiophilic secretion), from distal tip down to the socket cuticle. **a** Close-up slightly below the terminal pore. **b** At mid-level of the stalk. **c** Directly below the base of gland stalk. **d**-**f** Subcuticular portion of several tightly aggregated epidermal glands with stalked ducts in longitudinal perspective. Cross sections of tarsus 2. **d** Secretory cells and a canal cell. The socket cuticle surrounds the duct. **e** Close-up of the atrium-like structure and the proximate subcuticular aspect of the canal cell surrounded by the cuticle-lined distal part of the duct (magnified sector indicated by dashed box in (**d**)). Secretory cells probably associated with this gland are visible to the left. Next to the gland, epidermal cells are present. **f** Close-up of a gland similar to (**e**), but also showing that the duct continues into the gland stalk. Note also the complex, multilayered socket cuticle. Abbreviations: **at**, atrium-like structure, **cc** canal cell, **cu** cuticle, **du** duct, **epc** epidermal cell, **gr** secretory granule, **il** inner, fibrous layer, **in** cuticular intima, **mv**, microvilli, **ml** median, lamellated layer, **nusc** nucleus of a secretory cell, **rER** swollen cisternae of the rough endoplasmatic reticulum, **sc** secretory cell, **sl** extremely electron-dense outer layer (secretion layer), **scu** socket cuticle

TEM observations reveal that the glandular tissue in the ultimate leg of *H. subterraneus* consists of numerous 4-cell-units, which are tightly aggregated. Based on their cellular composition, proportion, and pattern of extracellular compartments, epidermal glands with stalked ducts belong to the class of recto-canal glands (sensu [22]). Each gland unit includes a canal cell, an intermediary cell, and two syntypic secretory cells (Figs. 8 and 9d) that form and surround the secretion-discharging extracellular space, which consists of two narrow, tubular compartments: the proximal, non-cuticularized reservoir and the distal, cuticularized duct (canal). The wall of the stalked duct is composed of four layers: (1) an outer, extremely electron-dense layer of varying thickness (Fig. 9a, b; sl), (2) a median, lamellated layer of moderate electron-density (Fig. 9a–c; ml), (3) an inner, non-lamellated, but diffusely fibrous layer being lesser electron-dense than the median layer and especially strong in the socket region (frayed in the distal region of the stalk) (Fig. 9b, c; il), and (4) a thin intima lining the duct (Fig. 9c–f; in). The outer layer is also present on interstitial cuticular areas, between the stalked ducts (Fig. 9d–f), filling up small depressions, and most likely equivalent to interfaces of the scutes. Thus, this layer is considered either leftovers of former released secretion, which was produced by the same type of gland or surrounding flexo-canal epidermal glands scattered in the glandular epithelium. Consequently, this layer is not an integral component of the stalked duct cuticle itself. The median and inner layers most likely represent cuticle secreted by interstitial epidermal cells, whereas the cuticular intima is produced by the canal cell. Apically, the duct opens via the pore at the tip of the stalk. The duct was always found filled with secretion (Fig. 9a–f).

**Fig. 10:**
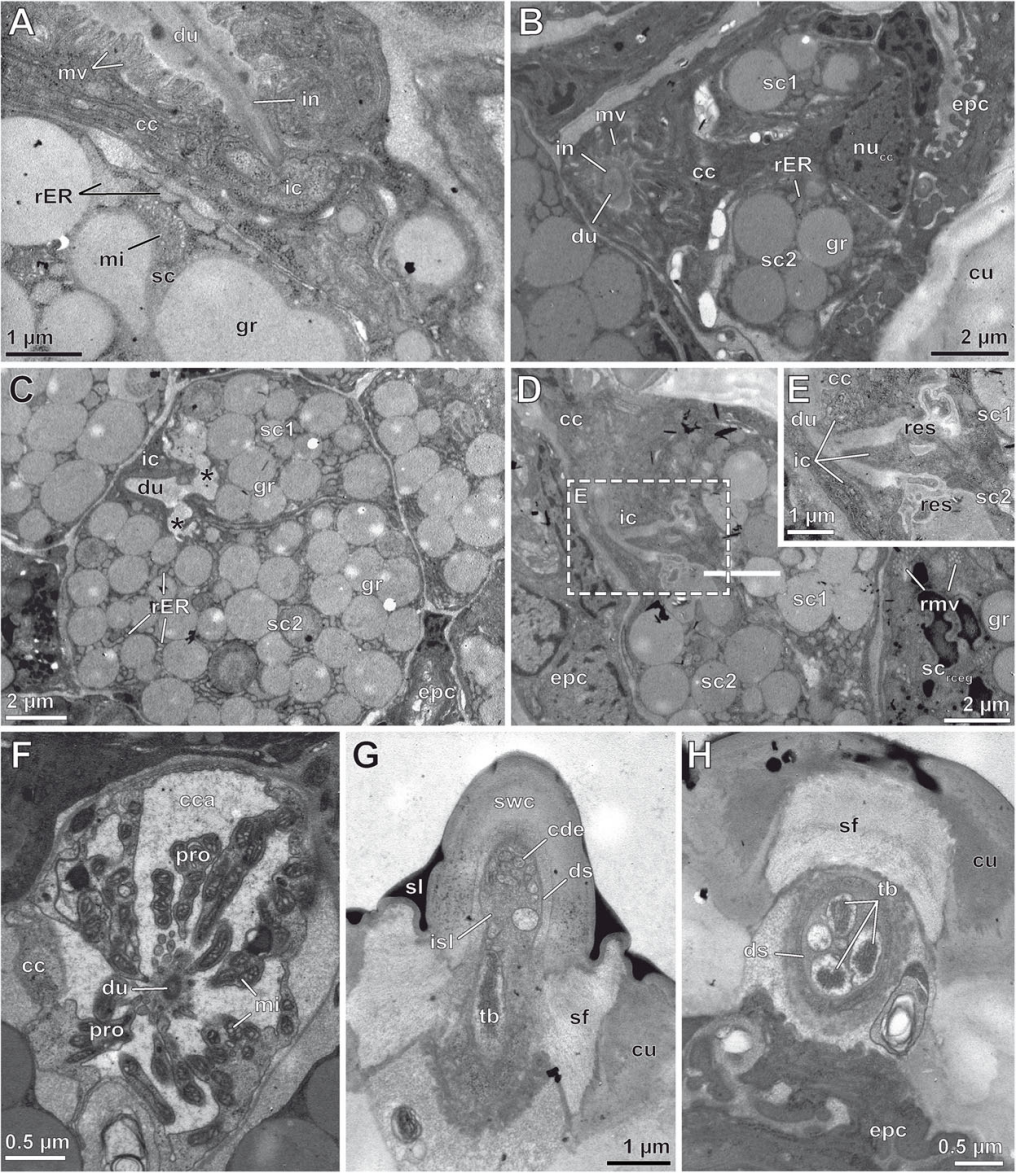
Ultrastructure of epidermal glands with stalked ducts (intermediary and secretory cells, **a**-**e**), small solitary epidermal glands (**d**, **f**), and sensilla trichodea (**g**, **h**) located on the male ultimate leg of *Haplophilus subterraneus* as depicted from TEM. **a** Transition zone of canal cell and intermediary cell. Note that the duct surrounded by the canal cell is lined by a cuticular intima. Cytoplasmic details of a secretory cell (not necessarily of the same gland) are visible to the lower left. **b** Tangential section of both secretory cells (reservoirs not cut) encompassed by canal cell with extremely electron-dense and barely contrasted cytoplasmic content, the cuticle-lined duct is cross-cut surrounded by microvilli formed by canal cell. Intermediary cell not visible. **c** Apex of both strongly granulated secretory cells more proximal than in (**b**). Note that both reservoirs (asterisks) converge to form the duct (non-cuticularized at this level). **d** and **e** Section of apical region of both secretory cells located between (**b**) and (**c**) demonstrating the dichotomic structure of the most proximal duct region (region magnified in inset (**e**), marked in (**d**) by dashed box). Cytoplasm of intermediary cell barely distinguishable from surrounding cells. **f** Canal cell of a solitary flexo-canal epidermal gland in cross-section. Note the extensive system of microvilliform projections, which project through the expanded central cavity and make terminate at the outer surface of the duct wall. **g** and **h** Sensillum trichodeum slightly above (**g**) and below (**h**) the socket level. Sensillum shown in (**g**) shows the basal aspect of the shaft wall cuticle, 10 small chemoreceptive dendritic outer segments, and a single tubular body. Sensillum shown in (**h**) included only two chemoreceptive dendritic outer segments, but the full set of three tubular bodies. Abbreviations: **cca** central cavity, **cu** cuticle, **cc** canal cell (of a flexo-canal epidermal gland), **cde** chemoreceptive dendritic outer segments, **ds** dendritic sheath, **du** duct, **epc** epidermal cell, **gr** secretory granule, **ic** intermediary cell, **in** cuticular intima, **isl** inner sensillum lymph space, **mi** mitochondrion/mitochondria (cristae type), **mv** microvilli, **nu**_**cc**_ nucleus of a canal cell, **pro** microvilliform projection, **rER** swollen cisternal of the rough endoplasmatic reticulum, **res** reservoir, **rmv** ring of microvilli (secretory cell), **sc**_**rceg**_ secretory cell of a recto-canal epidermal gland, **sc** secretory cell (of epidermal gland with stalked duct), **sc**_**1**_ secretory cell 1 (of epidermal gland with stalked duct), **sc**_**2**_ secretory cell 2 (of epidermal gland with stalked duct), **sf** socket fibres, **sl** extremely electron-dense outer layer (secretion layer), **swc** shaft wall cuticle, **tb** tubular body/bodies

The canal cell surrounds the cuticle-lined duct (Figs. 8, 9d–f and 10a; cc) that, mostly round in cross-profile, continuously decreases in diameter along the stalk up to the terminal pore (Fig. 9a–c). Immediately below the socket, the duct widens into a voluminous, drop-shaped atrium, which is bordered by infoldings of the inner, non-lamellated cuticle (Figs. 8 and 9d, e; at). Below the atrium and the leg’s surface cuticle, the duct tapers and is surrounded by the cuticular intima and a circumferential array of microvilliform processes of varying lengths, projecting towards the cuticle of the duct lumen (Figs. 8 and 10a, b; mv). The soma of the canal cell is crammed in the interspace of the apices of both subjacent secretory cells; its nucleus contains moderate to low portions of heterochromatin (Figs. 8 and 10b). The cytoplasm is extremely electron-dense resulting in a contrast too poor to obtain comprehensive insights of cytoplasmic organelles. Best recognizable organelles were scattered ER cisternae and mitochondria of the cristae type. The intermediary cell is very small, poorly supplied in membrane-delimited organelles, and exhibits generally a highly electron-dense cytoplasm (Figs. 8 and 10a, d, e; ic). The most proximal part of the duct, formed and surrounded by the intermediary cell, was observed to be widely free of a cuticular intima, but could not be convincingly documented on TEM micrographs due to lacking contrast at the interface of duct and cytoplasm. The intermediary cell forms a collar around the apices of both secretory cells as well as their tubular reservoirs (Figs. 8 and 10a, d, e). Both reservoirs converge to form the common, initially non-cuticularized duct at the level of the intermediary cell (Figs. 8 and 10d, e; res). The two secretory cells are densely granulated, equally sized, and bottle-shaped. Despite belonging to the same type, they are termed here as secretory cell 1 and 2 (Fig. 10b–e; sc). Each secretory cell bears a small but diversified reservoir, the shape of which is caused by coherence and dynamic turnover of slender and short apical microvilli and infoldings of the apical membrane, left over by former exocytosis events (Figs. 8 and 10c–e). Both secretory cells are rich in polymorphic secretory granules that are 0.1–0.3 μm in diameter and contain a homogenous, moderately to highly electron-dense matrix free of fibrillous profiles (Figs. 9d-f and 10a–e). In both secretory cells, the nucleus is located in the proximal part. It contains low portions of heterochromatin (Fig. 9d; nu). Membrane-bound inclusions are tightly arranged around many secretory granules. The majority of these inclusions are coated by minute, extremely electron-dense spherules, which strongly resemble ribosomes. Thus, we identify these inclusions as swollen cisternae of a highly active smooth and rough ER (Figs. 8, 9d, e and 10a–c; rER). Some micrographs reveal that the cisternae abut and feed their content directly into the secretory granules. Other organelles frequently observed are Golgi stacks with minute, often convoluted cisternae, which are also squeezed into the tiny interspaces between the tightly secretory granules. One to two interstitial epidermal cells are present between two adjoined epidermal glands thus forming a thin sleeve around each gland unit. However, this wrapping becomes only apparent in the distal part of the gland, since the nuclei of the interstitial epidermal cells are usually displaced to a confined area immediately below the cuticle (Figs. 9e, f and 10d). Moreover, the presence and number of interstitial epidermal cells is indicated by 1–2 scutes separating the sockets of the gland stalks on the surface cuticle.

Frequently, solitary recto- and flexo-canal epidermal glands are detectable within the glandular epithelium of the ultimate leg of *H. subterraneus*. These small epidermal glands can be distinguished by their pore structure (simple vs. walled), the course and compartmentalization of the duct as well as by the degree of infolding and microvillar differentiation of the apical membrane of the canal cell (Figs. 8 and 10f). In *H. subterraneus*, flexo-canal epidermal glands consist of three cells: a granulated secretory cell, a granulated intermediary cell, and a canal cell (Fig. 8). The apex of the canal cell always is deeply invaginated and convoluted. The apical membrane projects numerous microvilliform processes riddled with mitochondria of the cristae type (Figs. 8 and 10f; pro). The cuticularized duct is thin, convoluted, and always contains a mass of high electron-density, most probably secretion. The reservoir of the secretory cell is narrow and diversified in numerous canaliculi. The cellular set-up generally corresponds with that given in the semischematic drawing by Müller et al. [21]. Recto-canal epidermal glands contain three cells: a voluminous, drop-shaped, and strongly granulated secretory cell, a canal cell, a thin intermediary cell, and an elongated canal cell. The apex of the secretory cell shows a coniform process, which is surrounded by a palisade-like formation of elongated microvilli (Figs. 8 and 10d; rmv). The cellular architecture corresponds well to the semischematic reconstruction provided by Müller et al. [22].

Trichoid sensilla are flexibly inserted into the cuticle by a socket structure. The base of the sensillum shaft is suspended from surrounding cuticle by lesser electron-dense socket fibers (Fig. 10g, h; sf). The sensory portion consists of three mechanoreceptive neurons. Their dendrites (=outer dendritic segments) remain short and attach to the flexible socket via three tubular bodies (Fig. 10h; tb). In addition, elongated outer dendritic segment of 3–10 chemoreceptive neurons invade the sensillum shaft and project towards the terminal pore (Fig. 10g, h; cde). The outer dendritic segments are embedded into the sensillum lymph space encompassed by the dendritic sheath.

### Ultimate legs of other geophilomorph species possess similar epidermal glands

**Fig. 11:**
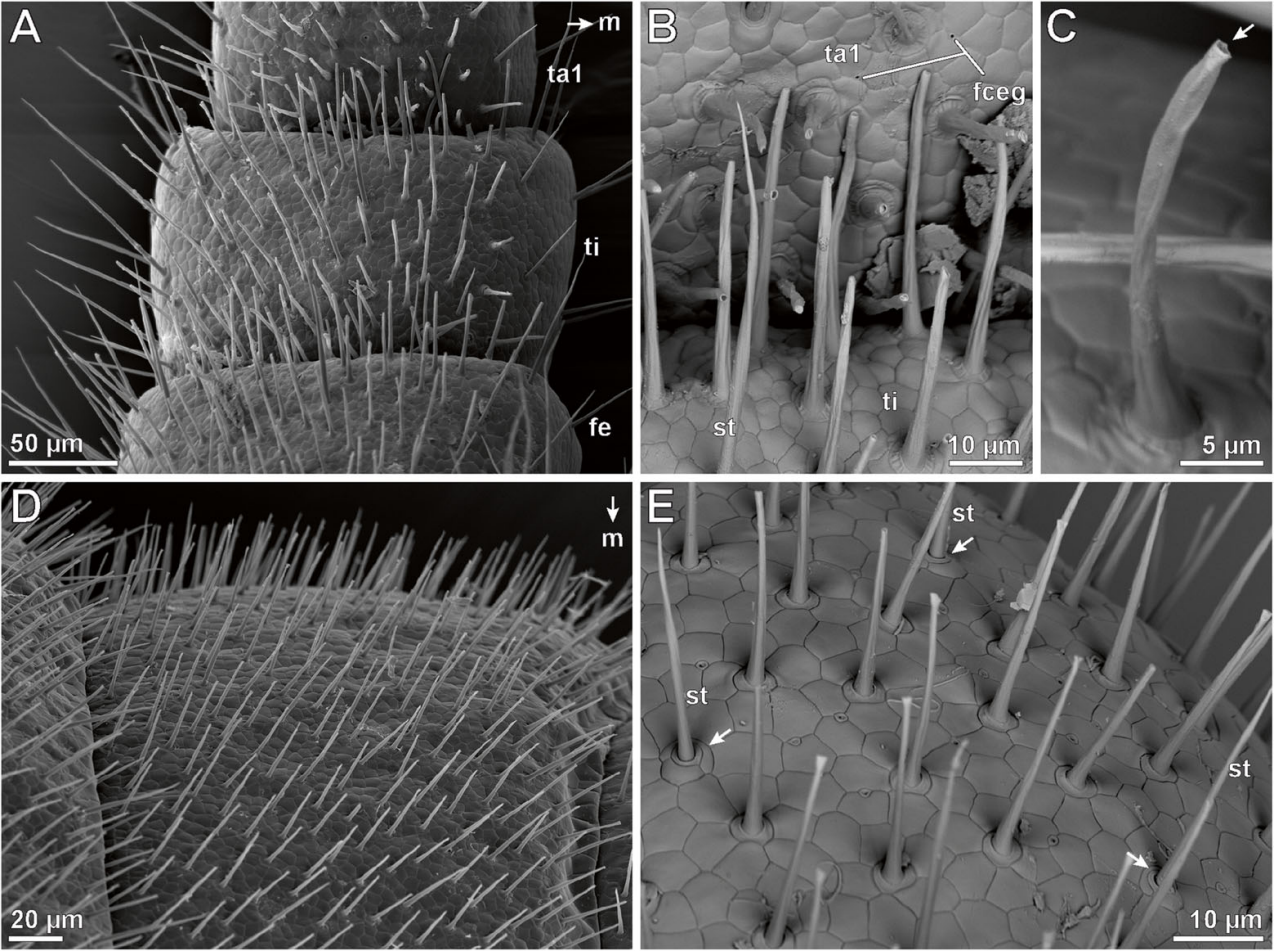
Epidermal glands with stalked ducts in *Strigamia maritima* and *Henia vesuviana*. **a** Ultimate leg podomeres of male *S. maritima*. View from ventral; medial to the right. The ultimate leg is intensively covered with stalked ducts of numerous epidermal glands, except for the medial side, which is mostly smooth. **b** A group of stalked ducts of epidermal glands at the transition of tibia and tarsus 1. Due to usage of backscattered electrons for SEM analysis, the large pores are clearly visible. Note the single trichoid sensillum at the bottom left (with pointed apex). Stalked ducts of epidermal glands display a round socket with radially ribbed surface structure. Frequently, small pores of flexo-canal epidermal glands are present. **c** Magnification of a stalked duct (backscatter electron analysis). The arrow points to the large pore. **d** Ultimate leg podomeres (lateral tibia) of male *H. vesuviana*. View from ventral; medial to the bottom. Stalked ducts of numerous epidermal glands densely cover the ultimate leg. **e** Detail of tarsus 2 (backscatter electron analysis). Only few trichoid sensilla are present, but clearly detectable by their socket structure (arrows). Stalked ducts of epidermal glands possess a round socket. In this species, the tips of the stalks appear slightly compressed. Frequently, small pores of flexo-canal epidermal glands are present. Abbreviations: **fceg** pores of flexo-canal epidermal glands, **fe** femur, **m** medial, **st** sensillum trichodeum, **ta1** tarsus 1, **ti** tibia

To check whether the occurrence of epidermal glands with stalked ducts on the ultimate leg pair is restricted to *Haplophilus subterraneus* (Himantariidae) or whether it might be a general feature of geophilomorph centipedes, two further species from different families were examined using SEM. In male *Strigamia maritima* (Linotaeniidae) and *Henia vesuviana* (Dignathodontidae), ultimate legs are also heavily covered with gland stalks and few trichoid sensilla; the medial faces are nearly free of any cuticular protuberances (Fig. 11a, d). In both species, stalked ducts of epidermal glands can easily be distinguished from shafts of trichoid sensilla by their socket structure (glands with round socket, sensilla with sickle-shaped scute) (Fig. 11b, e; st). The length of gland stalks is approx. 25–35 μm in *S. maritima* and approx. 25 μm in *H. vesuviana*. In *S. maritima*, the socket is radially ribbed (Fig. 11b), in *H. vesuviana* the socket is smooth (Fig. 11e). Trichoid sensilla possess a pointed apex, while stalked ducts of epidermal glands in *S. maritima* exhibit a large terminal pore (Fig. 11b, c). In *H. vesuviana*, the terminal pore often appears bilaterally compressed (Fig. 11e). In both species, the ratio of epidermal glands with stalked ducts and trichoid sensilla is roughly estimated 10:1. In both species, frequently small pores of flexo-canal epidermal glands are present (Fig. 11b, e). Both species possess a small claw (pretarsus) at the ultimate leg; a terminal invagination is absent (not shown).

## Discussion

### Secretory ultimate legs in geophilomorph centipedes

The presence and identity of ultimate leg associated epidermal glands with stalked ducts is a new discovery for geophilomorph centipedes and myriapods [22–24]. Previous descriptions of ultimate legs of geophilomorph species solely referred to a “hirsute” appearance (e.g. [7, 12]). Most taxonomic studies rarely utilized high magnification scanning electron microscopic images to document cuticular structures (e.g. [25–28]), but rather depict characteristics at low magnification or schematic drawings (e.g. [29–33]). Commonly, focus structures are the coxal pores (compare Fig. 1b, c), and characteristics of podomere proportions and the pretarsal claw. As the presence, number, and distribution of coxal pores on the ultimate leg coxa are accepted to be species-specific characters in Geophilomorpha [34], there might have been no explicit need in further exploring the specific morphology of the ultimate leg telopodite. Our analysis not only revealed the existence, but also common patterns of epidermal glands with stalked ducts on the ultimate legs of three systematically distant species of geophilomorph centipedes. Based on this knowledge, we are now able to re-assess scanning electron micrographs of previous studies dealing with ultimate legs of further species of Geophilomorpha. For instance, long and short cuticular structures (sensilla and presumably stalked ducts of epidermal glands) are evident on the ultimate leg femur, tibia, and both tarsi in female *Geophilus hadesi* [27]. Stalked ducts of epidermal glands with round socket and large apical pore are detectable on tarsus 2 of the ultimate leg at higher magnification, interspersed between trichoid sensilla, which are distinguishable by the smaller sickle-shaped scute (compare their fig. 17, bottom left in [27]). Likewise, SEM analyses of several species of the genus *Aphilodon* (Aphilodontidae), reveals stalked ducts of epidermal glands present on the ultimate legs (compare [28]). At least in some higher magnified images (figs. 73G, 74E, 81E, 93C, 112B in [28]), a clear separation of stalked ducts of epidermal glands and sensillar shafts is possible. Verhoeff [7] observed in *Geophilus carpophagus* (Geophilidae) that male ultimate legs are swollen and besides longer sensilla (“Tastborsten”) possess a furry surface of small bristles, however, without giving any images. These indications and their presence in the two additional examined species in our present study (Fig. 11) may indicate that epidermal glands with stalked ducts could be a ubiquitous feature on the ultimate legs of geophilomorph centipedes. As differentiation requires detail scale resolution under SEM, the true diversity and distribution of epidermal glands with stalked ducts on the ultimate leg of geophilomorphs is still not understood. Currently, shaft-like cuticular projections are mostly unified under the neutral term ‘setae’ (compare [15]). It would be desirable if future taxonomical studies focus specifically on the presence and characteristics of epidermal glands with stalked ducts using the set of identifying characters (large terminal pore, socket structure and morphology of surrounding scutes) as defined in this paper. Such future studies will certainly test our hypothesis that this newly described type of epidermal glands is common and may thus carry substantial resolving power for both phylogenetic and taxonomic investigations.

Even though progresses have been made in recent years, interrelationships of and subdivisions among some geophilomorph subclades (within Himantarioidea and Geophiloidea) have not been sufficiently resolved yet (see combined morphological and molecular analysis in [35]). The results of our study, and in particular the unexpected finding of ‘glandular hairs’ instead of sensory ones, may account for a striking example for how deceptive morphological descriptions of epidermal organs may be if based on non-invasive morphological analysis techniques alone, like SEM for instance. In fact, arthropod species descriptions or comparative morphological work with taxonomic or systematic focus usually lack aspects of invasive morphology. Nevertheless, the usage of microCT-generated anatomical data has become more and more popular in taxonomy and their holistic benefit for centipede taxonomy was demonstrated lately (e.g. [36, 37]). However, the true nature of these gland stalks would not have been fathomed by the aid of microCT analysis alone because necessary ultrastructural resolution is not yet met, but first approaches demonstrate the huge potential of this method (e.g. [38–40]). Epidermal glands with stalked ducts would certainly have slipped our attention if paraffin or semithin sectioning and/or high-detail TEM level examination had not been applied. Therefore, we strongly recommend that taxonomic investigations on arthropods should also gather anatomical data, at least as far as cuticular structures and their proper identification are concerned.

### Epidermal glands in Chilopoda – structural disparity and evolutionary scenarios

Epidermal glands with stalked ducts have never been recorded on centipede legs before and even if one widens the scope towards solitary exocrine epidermal glands of arthropods, it is difficult to find any equivalents. A potential candidate for comparison amongst Chilopoda could be epidermal exocrine glands with bottle-shaped shafts located at the border between the labral and clypeal part of the epipharynx in Lithobiomorpha [41, 42]. Both, the position and arrangement of the bottle-shaped shafts as well as the enormous size of the terminal pore indicate the glandular function of these structures, but this assumption needs to be confirmed by histological sections and, if applicable, also TEM observations. As a second, more comprehensively documented equivalent one may discuss the interommatidial exocrine glands of the water strider *Aquarius remigis*, as in this species the pore region is stretched above the cuticular surface level to form a ‘nail-headed structure’ [43], however, much shorter than described here for *H. subterraneus*. There are no striking similarities to more complex glands assumed to be involved in the production of silk threads in some myriapods such as ‘spinnerets’ of Symphyla [44] or the silk glands of bristly millipedes (Penicillata, Diplopoda) that are associated with the penes [45]. Based on specific anatomical features, such as the upright duct diversified by local widenings (i.e. atrium, reservoirs), epidermal glands with stalked ducts represent a new variant of the class of recto-canal epidermal glands known from all centipede taxa [22]. As opposed to the mostly tricellular flexo-canal epidermal glands [21], cell numbers in solitary or aggregated recto-canal epidermal glands are known to vary from 3 to 6 between species [22]. In particular, numbers vary in canal cells (1–3) and secretory cells (1–2). However, ultrastructural similarities and the presence of a single intermediary cell led to the hypothesis that recto-canal epidermal glands are homologous across centipedes, regardless of their cellular complexity and arrangement [22]. In investigated Geophilomorpha (*Strigamia maritima*, *Stigmatogaster dimidiatus*), recto-canal glands scattered in the head epidermis include only three cells: a canal cell, an intermediary cell, and a secretory cell. In syntypic aggregated sternal (defensive) glands, an apomorphic character of the Adesmata [35], two different types of secretory cells are present: a small and granulated cell with a narrow reservoir framed by microvilli (type-1 cell) and a much bigger, non-granulated cell with an extended tubular reservoir (type-2 cell) [46]. Such recto-canal epidermal glands with two secretory cells of extremely unequal size are widespread in the epidermis of lithobiomorph, craterostigmomorph and scolopendromorph centipedes [46, 47]. Huge reservoir spaces and widened duct compartments, numerous microvilliform processes invading the duct aiding extrusion, as well as a usually elaborated pore closure apparatus are features assumed to enable a rapid discharge of large amounts of secretion of various kinds (i.e. venom, deterrents, anti-adhesives) [22, 46]. The same basic release mechanisms and function may generally apply for the glands with stalked ducts of geophilomorph centipedes.

Typologically, the epidermal glands with stalked ducts of *H. subterraneus* represent a further variant of recto-canal epidermal glands, since the two secretory cells are of equal size and type. This variant is recorded in geophilomorphs for the first time. A similar configuration, however, was reported from tightly aggregated units of the maxillary organ gland of *Scutigera coleoptrata*, supporting the cleaning action of the maxillary organ [48]. Other, but not overly frequent examples for scattered epidermal glands with two syntypic and isometric secretory cells are known from flexo-canal epidermal glands in both Chilopoda (interommatidial glands of *Lithobius forficatus* [49]) and Diplopoda (postgonopodial glands of *Glomeris marginata* [50]*,* anal glands of *Rhapidostreptus virgator* [51]). Unless developmental studies are carried out, it is surely premature to speculate on the evolutionary origin of recto-canal epidermal glands with stalked ducts and two syntypic and isometric secretory glands in Geophilomorpha, but two hypotheses seem to be possible: (1) Evolution from solitary 3-cell recto-canal epidermal glands. In this scenario, the second secretory cell would have been recruited by duplication. Both, the aggregation of units and acquisition of the gland stalk would have been the result of secondary evolution. (2) Evolution from aggregated 4-cell recto-canal epidermal glands. This scenario implies that a precursor structure, perhaps similar to sternal glands [46], was transformed by having lost the type-2 secretory cell but retained and secondarily duplicated the type-1 secretory cell. Transformation processes then also reshaped the duct system (i.e. diminishment of the atrium, etc.).

### Sensory organs on the ultimate legs

Based on his histological analysis in *Himantarium samuelraji*, Rajulu [11] described two different sensory organs on the ultimate legs. Type 1 organs are exclusively present on the ventral sides of the ultimate leg tarsi and possess a thin cuticular plate that is slightly depressed below the level of the surrounding cuticle. Type 2 sensory organs are typical trichoid sensilla and electrophysiological experiments indicated a mechanosensory function [11]. It has to be noted here that the study by Gowri and Nageswaran [52] presents exactly the same text and data as that of Rajulu [11] by only stating a different species from another location and thus has to be considered as plagiarism. In *Haplophilus subterraneus*, type 1 organs were not detected, neither in histology nor in TEM analysis. It is possible that they might represent the aggregated epidermal glands, as the depicted cluster of cells could resemble the cluster of secretory grana of the secretory cells (compare similarities of fig. 1 in [11] and Fig. 9d). However, without further investigations a comparison is premature. Nonetheless, it is surprising that Rajulu [11] did not mention the presence of glandular tissue in the ultimate legs.

The types of trichoid sensilla on the ultimate legs of *H. subterraneus* are similar to those described for other centipede species. The small sickle-shaped scute, the slightly helically ribbed shaft, and the presence of an apical pore are features commonly present in centipede sensilla trichodea [53–58]. Smaller sensilla microtrichodea with the same characteristics are present in pleurostigmophoran centipedes [55, 56, 58–60]. Based on the presence of tubular bodies, a relatively high number of outer dendritic segments and an apical pore, sensilla trichodea are bifunctional and able to detect both chemical and mechanical stimuli. In addition, the cones at the tip of the ultimate leg tarsus 2 in *H. subterraneus* (Fig. 5a) likely represent sensory structures, hence we suggest the neutral term ‘sensory cone’. As ultrastructural data on their cellular anatomy are not available at present it is not entirely clear, whether these cones are equivalent to so-called ‘spinous tubercles’ (non-articulated, stout and rounded cuticular structures at the ultimate leg tip of several geophilomorph species [13, 15]). An association with sensilla brachyconica (non-articulated, without pores) from the antennal tip in Geophilomorpha and Scolopendromorpha [55, 61, 62] is conceivable. As a similar cone with circular socket that possesses a tangential groove is also present on the locomotory leg 10 claw (not shown), this type of sensillum might be more widespread on the cuticle of Geophilomorpha.

### Ultimate leg anatomy – is tarsus 1 a tarsus?

By definition, an arthropod tarsus (distal most podomere of the telopodite) never possesses intrinsic muscles [63–66]. Locomotory legs in Geophilomorpha possess a single tarsus that is devoid of intrinsic musculature, only tendon flexor muscles project from the tibia into the proximal area of the tarsus [65, 67]. Ultimate legs in Geophilomorpha are characterized by two tarsi: tarsus 1 (synonyms: proximal tarsus, basitarsus or tarsus) and tarsus 2 (synonyms: distal tarsus, distitarsus or metatarsus) [15]. Already Verhoeff [68] depicted and discussed intrinsic musculature in ultimate leg tarsus 1 of *Geophilus carpophagus* and *Mecistocephalus* sp. (tendon muscle 3 or km3 after [68], compare also fig. 7b in [4]). In addition to this muscle (Figs. 6a and 7d, e; tendon muscle, tmu), we found intrinsic muscles that, similarly to femur and tibia, project to the ventrodistal border of the following podomere. As the tendon is located far more centrally (compare Fig. 7e), these additional muscles do not attach to the tendon, but to the distal joint and might affect the leg’s ability to lift tarsus 2. This is in contrast to ultimate legs in e.g. *Scolopendra morsitans*, where two true tarsi (without intrinsic musculature) are present [69]. Verhoeff [68] argued that in geophilomorph ultimate legs the tarsus 1 actually represents the tibia that got fragmented into two podomeres: the pretibia and the tibia (see also discussion in [4]). In locomotory legs of *Orya barbata*, three tendon muscles attach each in prefemur, femur and tibia [65], which likely matches the situation in *H. subterraneus* ultimate legs (tarsus devoid of intrinsic musculature). Flexor musculature to the ventrodistal border is present from prefemur to the ventral tibia-tarsus-border (see fig. 46 in [65]). Thus, the equipment of intrinsic musculature differs between locomotory and ultimate legs (as there is an additional podomere present), but the origin of the presumed pretibia is not traceable by the actual equipment of musculature. Future developmental analyses might deepen these insights and lead to an updated terminology in ultimate leg podomeres. As our contribution only touches this topic partially, we do not propose to change the terminology of geophilomorph ultimate leg podomeres. However, there are strong arguments that tarsus 1 indeed represents the tibia or a part of it arisen from a split or duplication event.

## Conclusion

Based on our morphological analysis, a pronounced secretory as well as moderate sensory function of the ultimate legs in *Haplophilus subterraneus* has to be assumed. Based on the number of ca. 500 bifunctional (chemo- and mechanosensitive) trichoid sensilla per ultimate leg, it surely holds an elaborate sensory function. For comparison, locomotory leg 10 only houses 40–50 trichoid sensilla (not shown). The high abundance on tarsus 1 and 2 also confirms the general experiments by Rajulu [11] although proven in a different species. Thus, previous assumptions on the sensory capacity of ‘hairy’ ultimate legs are correct, although their main function was undetected. As the number of epidermal glands with stalked ducts is sexually dimorphic (higher in males), the function of ultimate legs might be primarily connected to reproduction. Sexual dimorphism of geophilomorph ultimate legs occurs in several species and is commonly associated with bigger (swollen) and/or intense ‘hairy’ legs in males [12, 14, 32, 70]. However, scientific data on mating behavior and spermatophore placement in Geophilomorpha is scarce [18, 71, 72]. Klingel [18] briefly described mating and indirect sperm transfer in *Geophilus flavus.* Males produce silky threads that should originate from the genital opening at the so-called spinneret/penis (‘Spinngriffel’), but no anatomical analysis of this structure is present and a context with silk production is speculative. During the spinning process, ultimate legs are constantly raised and finally a spermatophore is placed into the net. Thus, a production of a silk-like secretion in the ultimate legs associated epidermal glands is possible. Alternatively, an impregnation of the threads with e.g. pheromones seems possible as the female approaches the net only 3–4 h after courtship and spermatophore placement [18]. In favor of a silk-like secretion, one may argue that broken gland stalks frequently exhibit a filled canal. If a more volatile pheromone would be secreted, the process of chemical fixation with aldehydes and/or dehydration with ethanol would probably have led to extraction of this more soluble type of secretion from the ducts and surface cuticle. Besides a biochemical characterization of the secretion, the chemical transformation of the freshly released secretion needs to be further examined. However, the secretion of adhesive substances is common among centipedes. For instance, ultimate legs possess a defensive function in combination with an increased number of epidermal glands, e.g. the telopodal glands in Lithobiomorpha that secret a sticky substance containing proteins and lipids to entangle potential predators [4, 6, 8, 73]. In simulated attacks, geophilomorphs (including *H. subterraneus*) lift their heads and the posterior trunk [74]. However, secretion from the ultimate legs was not recorded. In summary: we do not know yet what these legs are used for exactly, but we will continue our research to reveal the true nature of ultimate leg secretion in Geophilomorpha. Prior to detailed functional discussions, substantial biochemical analyses have to be conducted to assess the specific composition of the secretion, complemented by an electron microscopic documentation of its post-release appearance.

## Methods

### Experimental animals

Adult male and female specimens of *Haplophilus subterraneus* (Shaw, 1789) were collected in Greifswald (Germany) under dead wood. Adult male specimens of *Strigamia maritima* (Leach, 1817) and *Henia vesuviana* (Newport, 1845) were obtained from a breeding culture at the University of Vienna. Specimens were identified using keys by Barber [12] and Rosenberg [75] and kept in separate terraria supplied with natural habitat and regular moistening.

### Photography

Several male and female specimens of *H. subterraneus* were anesthetized by cooling and analyzed using an Olympus Tough TG-4 camera and the BK PLUS Lab system (Dun Inc., http://www.duninc.com/bk-plus-lab-system.html) with a customized Canon MPE 65 mm 1-5x micro-photography lens mounted on a Canon 6D camera. Image stacks were captured with Adobe Lightroom and processed using Zerene Stacker under PMax value.

### Histology

For paraffin histology, two female and two male adult specimens of *H. subterraneus* were anesthetized and fixed in Bouin’s solution for 2 days (compare [76]). After several washing steps in PBS (phosphate buffered saline, 0.1 M, pH 7.4), dissected ultimate legs were dehydrated in a graded series of ethanol, incubated in a 1:1 solution of 96% ethanol and tetrahydrofuran (Carl Roth #CP82.1) for 2 h, pure tetrahydrofuran for 18 h, and in a solution of 1:1 tetrahydrofuran and paraffin (Carl Roth #6643.1) for 24 h at 60 °C. Finally, preparations were embedded in pure paraffin and sectioned (5 μm) with a motorized rotary microtome (Microm HM 360). Sections were stained with Azan according to Geidies [77] and mounted in Roti-Histokitt II (Carl Roth #T160.1).

For semi-thin sections, four female and four male adult specimens of *H. subterraneus* were fixed for 24 h in a solution of 10 parts 80% ethanol, 4 parts 37% formaldehyde and 1 part 100% acetic acid (FAE, compare [78]). After dissection and washing in PBS, specimens were post-fixed for 1 h in 2% OsO_4_ (in water) at room temperature and, following dehydration in a graded series of acetone, embedded in Epoxy resin (Araldite CY212; Agar Scientific #AGR1030) or in Agar Low Viscosity Resin Kit (Agar Scientific #AGR1078). Serial semi-thin sections (1 μm) were prepared with a Microm HM 355 S and a Leica EM UC6 and stained using 1% toluidine blue in a solution of 1% sodium tetraborate or Richardson’s stain [79]. Sections were analyzed with a Nikon Eclipse 90i and Nikon Eclipse Ni. Images were processed in Adobe Photoshop CC by removing the background as well as using global contrast and brightness adjustment features.

### Scanning and transmission electron microscopy

After anesthetization by cooling, three female and three male adult specimens of *H. subterraneus*, as well as two adult male specimen of *H. vesuviana* and *S. maritima* were fixed in FAE (see above). After dissection and dehydration in a graded series of ethanol, specimens were transferred to glass vials and cleaned in an ultrasonic bath. Samples were critical-point-dried using the automated dryer Leica EM CPD300 and mounted on copper wire (Plano #16067) or carbon-conducted tabs (Plano #G3347). For scanning electron microscopy, samples were sputter-coated with gold or gold-palladium and examined with a Zeiss EVO LS10 (Imaging Center of the Department of Biology, University of Greifswald) and a JEOL IT 300 (Core Facility Cell Imaging and Ultrastructure Research, University of Vienna) using detectors for secondary and backscatter electrons. Length of cuticular structures were measured at the Zeiss EVO LS10 (Zeiss SmartSEM software).

Pieces of ultimate legs of two female and two male adult specimens of *H. subterraneus* were incubated in fresh fixative solution modified after Karnovsky [80] containing 2.5% glutaraldehyde, 2.5% paraformaldehyde, 1.5% NaOH, and 1.5% D-glucose, buffered with PBS. After washing in several changes of PBS, post-fixation in 2% OsO_4_ solution was conducted at room temperature for 3 h, followed by dehydration in a graded series of ethanol and embedding in EmBed 812 epoxy resin (Science Services). Ultrathin sections (55–70 nm) were prepared using a Leica UCT and mounted on Formvar-coated slot grids (Plano #G2500C), stained with uranyl acetate and lead citrate for 4 min each, and examined with a JEOL JEM-1011 transmission electron microscope operated at 80 kV (General and Systematic Zoology, University of Greifswald). Digital micrographs were obtained by the aid of the mid-mount camera Megaview III (Soft Imaging System) using iTEM imaging software.

### microCT analysis

One female and one male adult specimen of *H. subterraneus* were anesthetized and fixed in Bouin’s solution overnight. Preparations were washed in 70% ethanol, dehydrated in a graded ethanol series, and incubated in a 1% iodine solution (iodine resublimated in 99% ethanol; Carl Roth #X864.1) for 12 h. Preparations were washed several times in pure ethanol and critical-point-dried. Finally, samples were fixed on insect pins with super glue. Scans were performed with a Zeiss Xradia MicroXCT-200 (Imaging Center of the Department of Biology, University of Greifswald) at 30 kV, 6 W, and 4 s exposure time resulting in a pixel size of ca. 0.96 μm (male specimen) and 40 kV, 8 W, and 1 s exposure time resulting in a pixel size of ca. 0.95 μm (female specimen). Tomography projections were reconstructed using the XMReconstructor software (Zeiss Microscopy) resulting in images stacks (TIFF format). All scans were performed by using Binning 2 (resulting in noise reduction) and subsequently reconstructed by using Binning 1 (full resolution) to avoid information loss. Image stacks were further processed using Amira 6.4 (Thermo Fischer) and Drishti 2.4 [81].

## Data Availability

The data generated and/or analyzed during the current study are available from the corresponding author upon reasonable request.
